# Inter-Rater and Intra-Rater Reliability of Return-to-Work Screening Tests for UK Firefighters Following Injury

**DOI:** 10.3390/healthcare10122381

**Published:** 2022-11-27

**Authors:** Liam Noll, Jason Moran, Adrian Mallows

**Affiliations:** School of Sport, Rehabilitation & Exercise Sciences, University of Essex, Colchester CO4 3SQ, UK

**Keywords:** return to work, firefighter, tactical athlete, functional capacity, physical assessment

## Abstract

The aim of this study was to assess the inter-rater and intra-rater reliability of a return-to-work (RTW) screening test to be used on UK firefighters following injury. The inter rater and intra-rater reliability of eight tasks involved in a screening test was used to assess readiness to RTW for UK firefighters following injury. These tasks included the following: (1) putting on and removing a breathing apparatus set (BA), (2) a ladder lift simulation, (3) a ladder carry simulation, (4) a light portable pump (LPP) lift and carry simulation, (5) a hose run, (6) a ladder climb with leg lock, (7) a casualty evacuation and (8) a confined space crawl simulation. The inter-rater reliability between each individual screening task was interpreted as Excellent (ICC = 0.94–1.00) for eleven (68.75%) of the screening task videos and as Good (ICC = 0.75–0.88) for five (31.25%) of the screening task videos. Intra-rater reliability was interpreted as Excellent (ICC = 1) for twenty-six participants (74.3%), Good (ICC = 0.76–0.88) for eight participants (22.9%) and Moderate for one participant (2.8%). Due to the reliability of this screening test, it allows conclusions to be made from the results which can inform a RTW decision for a firefighter.

## 1. Introduction

Musculoskeletal injuries can account for one-third of all workplace-related injuries [1,2]. Common causes include overexertion, contact with equipment, slips, trip and falls [2]. Many work tasks contain some risk of injury; however, the extent of these risks differs depending on the type of sector and job role [3]. The risk of a work-related injury increases for individuals with athletic occupations, including firefighters, military personnel, police officers and paramedics, whose job role requires higher physical demands; for example, heavy lifting, kneeling and crouching [4,5,6,7]. Of these injuries, more than 40% were musculoskeletal-related [4,8].

Following a musculoskeletal-related injury, assessing an individual’s readiness to return to work (RTW) can be complex; many factors need to be considered, including physical performance in relation to the work task demands [9,10]. An individual may believe that they are ready to RTW, but if they are unable to meet the minimum work-related physical demands, an increase to reinjury has been shown [11,12].

To assess physical performance in relation to work task demands, during recruitment of athletic occupations, a physical screening test is used to determine if individuals possess the minimum required aerobic fitness and muscular strength standards [13,14,15]. However, no such test exists to determine if an individual can meet the minimum standards after injury. For example, the physical screening test used for recruitment of firefighters does not include all tasks involved during operational duties, including hose running and ladder carry [1,16]. Instead, UK firefighter selection tests were designed to help identify applicants physically suited to roles within UK fire and rescue services [17] and then once employed, individuals are trained in more specific tasks related to their firefighting role [18]. If operational tasks are unable to be completed effectively in emergency situations, a firefighter could put themselves at risk of danger, their operational colleagues and members of the public [19].

To date, limited research exists for the effectiveness of RTW screening tests to reduce reinjury rates for individuals returning to work in an athletic occupation, for example a professional athlete [20,21,22,23,24]. No research has included athletic populations who are not professional athletes, for example firefighters [20,21,22,23,24]. To start to address this, a recent study provided consensus for the inclusion of tasks to be adopted into a screening test that could be used to assess a firefighter’s readiness to RTW following injury [1].

However, before any screening test can be used to assess readiness to RTW, its reliability must be determined [25]. The reliability of a screening test should be of important consideration especially in settings where decisions on an individual’s ability to perform job related tasks at the required level as based on interpretation of the results [26]. A reliable screening test ensures the same or compatible results across different assessments, regardless of when the test took place, the environment in which the test is conducted in, or the professional administering the test [25,27]. Without sufficient inter-rater and intra-rater reliability, any screening test holds little value in determining if an individual is ready to return to the demands of their job role [28].

The aim of this study was to assess the inter-rater and intra-rater reliability of a RTW screening test to be used on UK firefighters following injury.

## 2. Materials and Methods

### 2.1. Study Design

An inter-rater and intra-rater reliability study of eight tasks involved in a screening test was used to assess readiness to RTW for UK firefighters following injury. The eight tasks in the screening test were gained by consensus during a recent Delphi study [1] and include the following; (1) putting on and removing a breathing apparatus set (BA), (2) a ladder lift simulation, (3) a ladder carry simulation, (4) a light portable pump (LPP) lift and carry simulation, (5) a hose run, (6) a ladder climb with leg lock, (7) a casualty evacuation and (8) a confined space crawl simulation.

### 2.2. Participant Criteria

A purposive sample, of occupational health, fitness professionals or operational firefighters working within fire services in the United Kingdom (UK) was recruited to be participants. Purposive sampling aimed to capture experts within the fire service. All participants were currently involved in health and fitness assessments of operational firefighters. There was no requisite on the number of years a participant had worked within their role.

### 2.3. Recruitment

Participants were recruited from the National Fire Chiefs Council Fitness Advisers and Occupational Health online groups. The researcher (LN) emailed fitness advisors, occupational health managers, occupational health nurses, occupational health advisors and operational firefighter trainers who currently work for UK fire and rescue services, inviting them to participate in the study. The email included a hyperlink to the study website page and a participant information sheet (PIS). All participants were required to give their consent by answering the pre-study questions before progressing further in the study.

### 2.4. Sample Size

A priori power analysis was conducted to estimate the sample size required using G* Power software (version 3.1.9.4), Franz Faul, Germany [29]. The results estimated that a sample size of thirty-five would be required to establish inter-rater and intra-rater reliability (H0 = 0.00, H1 = 0.70, α = 0.05, single tail, power = 0.95) [30]. To allow for attrition, we increased this estimated sample size by 10% and rounded up to the nearest whole number [31,32], leaving a sample size of thirty-nine.

### 2.5. Data Collection/Testing Procedure

Participants were provided access to a website, created using the E-learning tool Moodle [33]. The website hosted videos of the screening tests were recorded in 1080p HD video at 60 frames per second using an iPhone 12 and were edited in iMovie [34]. The iPhone 12 was set up on a tripod at approximately two meters [35] from the individual being recorded, from a front view. Each screening test was recorded two times with predetermined outcomes, (1. Pass, 2. Fail). All participants were unaware of the predetermined outcome for each video. The scoring criteria were based on the current national firefighter guidance for correct technique required for the tests [17].

All participants were required to watch an online training video detailing the online screening criteria form (SCF) before completing any rating as part of this study. The online training video was created by one of the researchers (LN) by screen recording of a mock screening test rating using Microsoft Teams [36]. The mock screening test was different from the included screening tests to avoid any influence on participants rating. After viewing the online training video, all participants were required to complete a multiple-choice questionnaire based on the training video with 100% pass mark required to pass the training. If any participants had difficulties with the online training, they were able to contact one of the researchers (LN) via email for assistance. To ensure audio and video quality, a pilot test was undertaken by one of the researchers (LN).

Participants visually assessed the technique used in the video for each screening test using a score criteria (“Pass” or “Fail”). Scores were based on a participant’s judgment regarding technique throughout the task using the scoring criteria provided for each task as a reference (Appendix A Table A1).

For each participant, two rating sessions were performed with two weeks separating each session as used in previous reliability studies [25,26]. The measures obtained from both rating sessions were used to estimate inter-rater reliability. The initial and follow up testing measures from participants were used to estimate intra-rater reliability. All participants were blinded to other participants’ scores by viewing the videos of the screening test online individually. All participants were advised to prevent any communication about the screening videos and/or ratings between each other. All videos were required to be rated in one sitting.

### 2.6. Statistical Analysis

Descriptive data were used to characterise the participants using means with standard deviations (SD) where applicable using a Microsoft Excel spreadsheet. Scores from the participants were initially stored in a Microsoft Excel spreadsheet.

Inter-rater and intra-rater reliability was assessed using Intra-class Correlation Coefficients (ICC) [25]. For inter-rater reliability, a two-way random-effects model, mean of k raters, and absolute agreement (ICC(2,k)) was used. For intra-rater reliability, a two-way mixed-effects model, mean of k measurements, and absolute agreement (ICC(3,1)) was used. Interpretation of reliability results was based on the following criterion: Excellent reliability (>0.90), Good reliability (0.75–0.90), Moderate reliability (0.50–0.75) and Poor reliability (<0.50) [37]. All statistical analysis were conducted using Statistical Package for the Social Services (SPSS) version 27 for Windows [38].

## 3. Results

### 3.1. Participants

**Figure 1:**
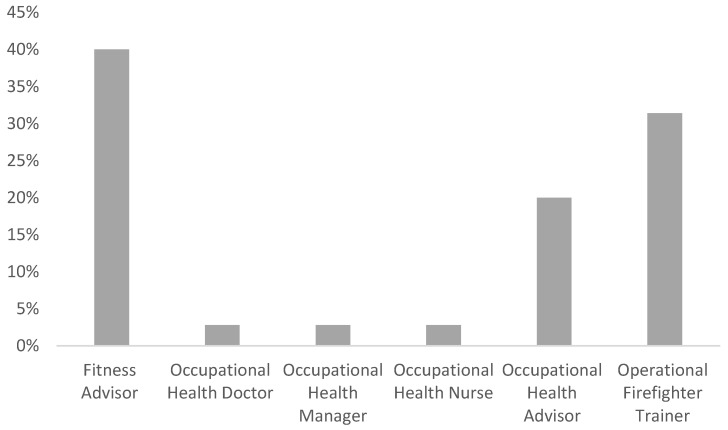
Bar Chart showing the job role of the participants.

**Figure 2:**
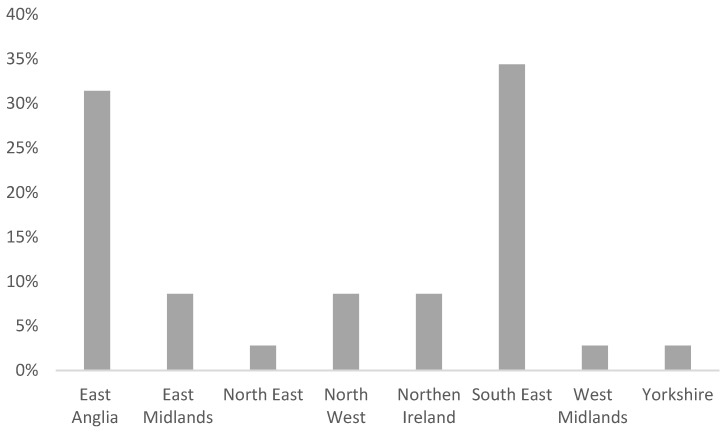
Bar chart showing the region representation in the United Kingdom of the participants.

Forty-two participants volunteered to participate in this study. Participants’ job roles within their service included fitness advisors (*n* = 14) (40%), occupational health doctor (*n* = 1) (2.8%), occupational health manager (*n* = 1) (2.8%), occupational health nurse (*n* = 1) (2.8%), occupational health advisor (*n* = 7) (20%) and operational firefighter trainer (*n* = 11) (31.4%) (Figure 1). From these, a total of thirty-five participants completed both online rating screening sessions (83.3% retention rate). There was representation from different fire and rescue services across the UK (*n* = 8) (Figure 2). Overall, the demographic of the participants was proportionally representative of the original invitation list. The mean age of the participants in this study was 40.34 + 9.02 years and the mean duration they had worked for their fire service was 12.40 + 8.11 years (Table 1).

### 3.2. Inter-Rater Reliability between All Screening Tasks

The inter-rater reliability between all screening tasks during both rating sessions was interpreted as Good (ICC = 0.77–0.79) (Table 1). For participants with 0–9 years of service, the inter-rater reliability between all screening tasks during both rating sessions was interpreted as Good (ICC = 0.76–0.81) and Good (ICC = 0.77–0.82) for participants with more than nine years of service (Table 2).

### 3.3. Inter-Rater Reliability between Each Individual Screening Task

The inter-rater reliability between each individual screening task was interpreted as Excellent (ICC = 0.94–1.00) for eleven (68.75%) of the screening task videos across both rating sessions. These tasks included, Ladder lift (Pass Video), Putting on a BA set (Fail Video), Ladder carry (Pass and Fail video), LPP lift and carry (Pass and Fail video), Hose run (Pass and Fail video), Casualty evacuation (Pass and Fail video) and Confined Space (Fail video) (Table 3).

Inter-rater reliability was interpreted as Good (ICC = 0.75–0.88) for five (31.25%) of the screening task videos across both rating sessions. These tasks included, Ladder lift (Fail video), Putting on a BA Set (Pass video), Ladder climb and leg lock (Pass and Fail video), Confined space (Pass video) (Table 3).

### 3.4. Intra-Rater Reliability

Intra-rater reliability was interpreted as Excellent (ICC = 1) for twenty-six participants (74.3%), Good (ICC = 0.76–0.88) for eight participants (22.9%) and Moderate for one participant (2.8%) (Table 4).

## 4. Discussion

Currently, no nationally agreed RTW screening test exists within UK fire services. To develop a nationally agreed test, previous research identified the tasks to be included [1]; however, the reliability was yet to be determined. This study aimed to assess the inter-rater and intra-rater reliability of a RTW screening test to be used on UK firefighters following injury. Results showed that the overall inter-rater reliability between all screening tasks was interpreted as Good (ICC = 0.77–0.79) for both rating sessions and the intra-rater reliability was interpreted between Moderate-Excellent (ICC = 0.63–1.00), with 97% of participants reliability being interpreted between Good-Excellent (ICC = 0.76–1.00).

Employers often reply upon screening tests assessing functional capacity to assist in determining an individual’s work capacity relevant to their specific job role [39]. The results from these screening tests can aid with the decision to allow an individual to return to their job role or help provide further rehabilitation interventions [39]. In addition, screening tests help provide a consistent method of assessment used within a workforce [39,40].

Similar studies assessing functional capacity set an ICC criterion of >0.75 for screening tests to be classed as “reliable” [41,42]. The inter-rater results from this study (ICC = 0.77–0.79) suggest that this screening test can be used to identify if a firefighter undertaking the RTW tasks passes or fails on a reliable basis. These data are important, as it is essential to have reliable screening methods when assessing a firefighter’s ability to complete operational tasks with the correct technique to determine their physical readiness to return to operational duties [43]. By identifying reliable RTW screening tests for the physically demanding role of a firefighter is key to help highlight those firefighters who are able to undertake their role effectively, therefore improving the safety of themselves, their colleagues and the public on their RTW [44]. Previous research concluded that reliability studies should focus on multiple raters of varying background and experiences [45,46]. This was achieved as thirty-five participants from eight fire and rescue service regions across the UK completed both of the required screening sessions. The results obtained were provided from professionals working across a range of occupational health, fitness and operational training departments, with an average of 12.40 + 8.11 years’ experience.

Intra-rater reliability is important in such measures because it determines the accuracy of an assessment where a single rater may make multiple assessments over time [47,48]. Our study showed that intra-rater reliability ICC ranged from 0.63–1.00 with 97% of participants achieving a reliability interpretation above the ICC criterion of >0.75 as shown in previous studies [41,42]. This suggests that the RTW screening test for firefighters following musculoskeletal injury used in this current study is suitable for repeated measures in assessing a firefighter’s readiness to RTW.

Reliability for repeated measures is especially important in assessing the consistency of the RTW screening test. A lack of consistency for RTW assessments following injury was perceived as a barrier amongst firefighters experienced during their RTW process [49]. Therefore, if this RTW screening test was used as good practice within UK fire and rescue services, it could potentially remove this barrier by adding trustworthiness to the RTW process and help to increase the consistency of the RTW assessment.

The online design of the RTW screening test used in this study increased the ease of access for participants, as they were able to complete the rating sessions for the RTW screening test on desktop or portable devices, including laptops, smartphones, and tablets. As a result, future practice could allow for this RTW screening test to be used in various locations across different fire and rescue services provided they have the required equipment for the screening test. This could increase the availability in RTW screening test appointments within fire and rescue services and as a result, help decrease potential waiting times for firefighters looking to return to their job role. Further research is needed to assess the validity of the use of this RTW screening test to help reduce firefighter reinjury rates in UK fire and rescue services.

### Strengths and Limitations

This study included experts from fire service fitness and occupational health departments as well as operational firefighters in the UK. Experts from fire and rescue services across the UK were invited to participate but this study participation did not include representation from every fire and rescue service in the UK. Nevertheless, those who did take part provided representation from a large range of UK fire and rescue services. The online approach helped reduce the impact on the participants. This study was focused on participants working for UK fire and rescue services. The online approach allows for representation from fire and rescue services internationally in future studies.

A training video and clear SCF provided the participants with the information required of what was required from them. The videos filmed, provided clear visual information for participants to decide if the video should be marked as pass or fail. The design of the website allowed the SCF and the assessment videos to be on the webpage. This allowed participants to use one screen/device and it could be completed on a computer desktop, tablet or mobile device.

## 5. Conclusions

The return-to-work screening test used in this study provided evidence that it has good inter-rater reliability (ICC = 0.77–0.79) and good-excellent intra-rater reliability (ICC = 0.76–1.00) for 97% of participants. Due to the reliability of this screening test, it allows conclusions to be made from the results which can inform a return-to-work decision for a firefighter. This return-to-work screening test provides a method for fitness and occupational health experts as well as operational trainers working for UK fire and rescue services to refer to when assessing the readiness of a firefighter to return to operational duties. If used, this screening test could increase the consistency of return-to-work process within UK fire and rescue services and add trustworthiness to the decisions made. Further research is needed on the validity of this return-to-work screening test in reducing reinjury rates within firefighters.

## Figures and Tables

**Table 1:** Overall inter-rater reliability of all screening tests for both rating sessions. ICC = Intraclass correlation coefficients, CI = Confidence interval.

Inter-Rater Reliability			
Rating Session	ICC_2,35_	95% CI	Interpretation
1	0.77	0.67–0.85	Good
2	0.79	0.71–0.87	Good

**Table 2:** Inter-rater reliability of all screening tests for both rating sessions based on years worked with the fire service. ICC = Intraclass correlation coefficients, CI = Confidence interval.

	Inter-Rater Reliability			
	Rating Session	ICC_2,35_	95% CI	Interpretation
0–9 years of service	1	0.76	0.66–0.85	Good
	2	0.81	0.72–0.89	Good
9+ years of service	1	0.77	0.68–0.86	Good
	2	0.82	0.75–0.89	Good

**Table 3:** Inter-rater reliability of each individual screening test video over two rating sessions. ICC = Intraclass correlation coefficients, CI = Confidence interval.

	Inter-Rater Reliability			
	Rating Session	ICC_2,35_	95% CI	Interpretation
Ladder lift (pass)	1	1.00	1.00–1.00	Excellent
	2	1.00	1.00–1.00	Excellent
Ladder lift (fail)	1	0.88	0.55–1.00	Good
	2	0.88	0.55–1.00	Good
Putting on a BA set (pass)	1	0.76	0.32–1.00	Good
	2	0.76	0.33–1.00	Good
Putting on a BA set (fail)	1	0.94	0.74–1.00	Excellent
	2	0.94	0.74–1.00	Excellent
Ladder carry (pass)	1	0.94	0.74–1.00	Excellent
	2	0.94	0.74–1.00	Excellent
Ladder carry (fail)	1	0.94	0.74–1.00	Excellent
	2	0.94	0.74–1.00	Excellent
LPP lift and carry (pass)	1	1.00	1.00–1.00	Excellent
	2	1.00	1.00–1.00	Excellent
LPP lift and carry (fail)	1	1.00	1.00–1.00	Excellent
	2	1.00	1.00–1.00	Excellent
Hose run (pass)	1	0.94	0.74–1.00	Excellent
	2	1.00	1.00–1.00	Excellent
Hose run (fail)	1	0.91	0.70–1.00	Excellent
	2	0.94	0.74–1.00	Excellent
Ladder climb and leg lock (pass)	1	0.82	0.43–1.00	Good
	2	0.82	0.43–1.00	Good
Ladder climb and leg lock (fail)	1	0.81	0.42–1.00	Good
	2	0.88	0.55–1.00	Good
Casualty evacuation (pass)	1	0.94	0.74–1.00	Excellent
	2	0.94	0.74–1.00	Excellent
Casualty evacuation (fail)	1	0.94	0.74–1.00	Excellent
	2	0.94	0.74–1.00	Excellent
Confined space (pass)	1	0.81	0.42–1.00	Good
	2	0.81	0.42–1.00	Good
Confined space (fail)	1	1.00	1.00–1.00	Excellent
	2	1.00	1.00–1.00	Excellent

**Table 4:** Intra-rater reliability between each rating session. ICC = Intraclass correlation coefficients, CI = Confidence interval.

Intra-Rater Reliability			
	ICC_(3,1)_	95% CI	Interpretation
Participant 1	1.00	1.00–1.00	Excellent
Participant 2	1.00	1.00–1.00	Excellent
Participant 3	0.63	0.37–0.80	Moderate
Participant 4	1.00	1.00–1.00	Excellent
Participant 5	1.00	1.00–1.00	Excellent
Participant 6	0.80	0.77–0.94	Good
Participant 7	0.81	0.66–0.91	Good
Participant 8	1.00	1.00–1.00	Excellent
Participant 9	0.76	0.56–0.87	Good
Participant 10	1.00	1.00–1.00	Excellent
Participant 11	1.00	1.00–1.00	Excellent
Participant 12	1.00	1.00–1.00	Excellent
Participant 13	0.76	0.56–0.87	Good
Participant 14	1.00	1.00–1.00	Excellent
Participant 15	0.88	0.77–0.94	Good
Participant 16	1.00	1.00–1.00	Excellent
Participant 17	1.00	1.00–1.00	Excellent
Participant 18	1.00	1.00–1.00	Excellent
Participant 19	0.88	0.77–0.94	Good
Participant 20	1.00	1.00–1.00	Excellent
Participant 21	1.00	1.00–1.00	Excellent
Participant 22	1.00	1.00–1.00	Excellent
Participant 23	0.88	0.77–0.94	Good
Participant 24	1.00	1.00–1.00	Excellent
Participant 25	1.00	1.00–1.00	Excellent
Participant 26	1.00	1.00–1.00	Excellent
Participant 27	1.00	1.00–1.00	Excellent
Participant 28	0.80	0.77–0.94	Good
Participant 29	1.00	1.00–1.00	Excellent
Participant 30	1.00	1.00–1.00	Excellent
Participant 31	1.00	1.00–1.00	Excellent
Participant 32	1.00	1.00–1.00	Excellent
Participant 33	1.00	1.00–1.00	Excellent
Participant 34	1.00	1.00–1.00	Excellent
Participant 35	1.00	1.00–1.00	Excellent

## Data Availability

Not applicable.

## Appendix A

**Table A1:** Screening Video Criteria Form (SCF).

Screening Test	Pass Criteria
Putting on and Removal of Breathing Apparatus Set	Firefighter squats behind the BA set with the top of the cylinder between their feet.
	Firefighter stands the set onto the cylinder bump stop so that it is in a vertical position
	Firefighter draws the set close to their body, bending the knees and keeping the spine in a neutral position whilst standing up.
	Firefighter places the right-hand shoulder strap over their right shoulder and then places left arm into the left shoulder strap.
	Firefighter fastens shoulder straps and then fastens waist belt buckle ensuring that the belt is not twisted.
	Firefighter fastens chest and waist clips then stands up straight.
Ladder lift simulator	Firefighter starts with an underhand grip on the bar with palms facing upwards.
	Firefighter bicep curls the bar, keeping back straight.
	Firefighter rotates their wrists one at a time so that the bar is now gripped with the bottom of their palms facing outwards.
	Firefighter shoulder presses the bar, without any assistance from the lower body, ensuring that the bar is above the designated yellow marker.
	Firefighter lowers the bar in a controlled manner back to chest height, changing wrists back over so that the bottoms of their palms are facing towards them.
	Firefighter lowers bar to the start position by extending their arm and places the bar into the rest position, bending their knees if required.
Ladder carry simulator	Firefighter starts with their feet flat on the ground and positioned between hip and shoulder width apart.
	Firefighter squats down and grasps the dumbbell in one hand.
	Firefighter lifts the dumbbell off the floor, by extending their knees and hips, until standing in a upright position. Firefighters back should maintain a rigid spine with a constant torso angle to the floor.
	Firefighter holds the dumbbell down by their side with a straight arm and proceeds to walk forwards, keeping an upright position.
	Once the firefighter has reached the required distance, they lower the dumbbell to the floor whilst maintaining a neutral spine, flexing the hips and squatting.
	Firefighter turns around and repeats the process, lifting the dumbbell with the opposite hand.
	Once the firefighter has reached the required distance, they lower the dumbbell to the floor whilst maintaining a neutral spine, flexing the hips and squatting.
Light portable pump lift and carry simulator	Firefighter starts with their feet flat on the ground and positioned between hip and shoulder width apart.
	Firefighter squats down and grasps the barbell with both hands.
	Firefighter lifts the barbell off the floor by extending knees and hips until they are in an upright position. Their back should maintain a rigid spine with a constant torso angle to the floor.
	Firefighter holds the barbell down in front of them with straight arms and proceeds to walk forwards keeping in an upright position.
	One the firefighter has reached the required distance, the barbell is lowered to the floor whilst maintaining a neutral spine, flexing the hips and squatting.
Casualty evacuation	Firefighter grasps the casualty, with both hands, by the carrying handle located at the back of the dummy’s head.
	Firefighter positions themselves body upright, back neutral and legs slightly bent.
	Firefighter drags casualty by walking backwards.
	Once the firefighter reaches the required distance, grasp on the carrying handle is released in a controlled manner.
Hose run	Firefighter places their foot on the hose and grasps the lugs with their hands.
	Firefighter lifts hose to shoulder height and holds it to the side of their body.
	Firefighter runs hose out until the end is reached and the female coupling is placed carefully on the ground.
	Firefighter runs back and underruns the hose.
Ladder climb and leg lock	Firefighter climbs the ladder and takes a leg lock.
	Firefighter releases their hands from the ladder, outstretches both arms to the side and looks over each shoulder.
	Firefighter regains hand hold on the ladder, removes their leg lock and descend the ladder to the ground.
Confined space crawl	Firefighter crawls on their hands and knees through the confined space.
	Once the firefighter reaches the end of the confined space, they turn around and make their way back to the start.
	The crawl should be completed in a calm and controlled manner by the firefighter.
